# Structure based drug discovery for designing leads for the non-toxic metabolic targets in multi drug resistant *Mycobacterium tuberculosis*

**DOI:** 10.1186/s12967-017-1363-9

**Published:** 2017-12-21

**Authors:** Divneet Kaur, Shalu Mathew, Chinchu G. S. Nair, Azitha Begum, Ashwin K. Jainanarayan, Mukta Sharma, Samir K. Brahmachari

**Affiliations:** 1 CSIR-Institute of Genomics and Integrative Biology, New Delhi, India; 2 Centre for Open Innovation-Indian Centre for Social Transformation, Bengaluru, Karnataka India; 3 Academy of Scientific and Innovative Research, New Delhi, India; 4 CSIR-Open Source Drug Discovery Unit, New Delhi, India; 5 Present Address: Indian Institute of Science Education and Research (IISER), Mohali, India

**Keywords:** Drug development, Drug resistance, *Mycobacterium tuberculosis*, Non-toxic targets, Structural biology, Systems biology

## Abstract

**Background:**

The problem of drug resistance and bacterial persistence in tuberculosis is a cause of global alarm. Although, the UN’s Sustainable Development Goals for 2030 has targeted a Tb free world, the treatment gap exists and only a few new drug candidates are in the pipeline. In spite of large information from medicinal chemistry to ‘omics’ data, there has been a little effort from pharmaceutical companies to generate pipelines for the development of novel drug candidates against the multi drug resistant *Mycobacterium tuberculosis*.

**Methods:**

In the present study, we describe an integrated methodology; utilizing systems level information to optimize ligand selection to lower the failure rates at the pre-clinical and clinical levels. In the present study, metabolic targets (Rv2763c, Rv3247c, Rv1094, Rv3607c, Rv3048c, Rv2965c, Rv2361c, Rv0865, Rv0321, Rv0098, Rv0390, Rv3588c, Rv2244, Rv2465c and Rv2607) in *M. tuberculosis*, identified using our previous Systems Biology and data-intensive genome level analysis, have been used to design potential lead molecules, which are likely to be non-toxic. Various in silico drug discovery tools have been utilized to generate small molecular leads for each of the 15 targets with available crystal structures.

**Results:**

The present study resulted in identification of 20 novel lead molecules including 4 FDA approved drugs (droxidropa, tetroxoprim, domperidone and nemonapride) which can be further taken for drug repurposing. This comprehensive integrated methodology, with both experimental and in silico approaches, has the potential to not only tackle the MDR form of Mtb but also the most important persister population of the bacterium, with a potential to reduce the failures in the Tb drug discovery.

**Conclusion:**

We propose an integrated approach of systems and structural biology for identifying targets that address the high attrition rate issue in lead identification and drug development We expect that this system level analysis will be applicable for identification of drug candidates to other pathogenic organisms as well.

**Electronic supplementary material:**

The online version of this article (10.1186/s12967-017-1363-9) contains supplementary material, which is available to authorized users.

## Background

Tuberculosis (Tb), caused primarily by *Mycobacterium tuberculosis* (Mtb), is a major world-wide disease affecting millions of individuals every year, with high mortality rates. The World Health Organization’s goal of ‘End-Tb Strategy’ and the United Nation’s Sustainable Development Goals (SDGs) (Goal 3; target3) lay the roadmap for achieving a global goal of ending the Tb epidemic by 2030. The unmet medical need followed by the recent emergence of multi drug resistant (MDR) and extreme drug resistance (XDR) strains of Mtb [1, 2] continues to be a roadblock in achieving this goal‬‬ [3–5]. There are very few drugs for treating Tb (MDR/XDR) and various reasons exist for the lack of new medicines, including the lack of funding in Pharmaceutical Research & Development for such neglected diseases. The prohibitive cost of drug development has been attributed to poor target selection and due to this, 87% of the late-stage failures can be avoided, as they show poor efficacy and side effects [6]. In addition, the market size of Tb drugs is also low and not attractive to multi-national companies.

In the present situation, understanding of the complex biological responses or the systems biology of an organism is highly significant to improve and fasten the process of drug development by reducing the failure rates. Methods of selective chemical tailoring of molecules based on the knowledge of existing lead compounds against Mtb, which can also address the emerging resistance issues, has the potential of fueling the Tb clinical pipeline. In order to minimize the chances of failure and cost of Tb drug discovery, innovative approaches for designing newer chemical entities, using data intensive in silico approaches, involving experimentally validated data is the need of the hour. Keeping this in mind, the Open Source Drug Discovery (OSDD) project was initiated to facilitate the data-driven drug discovery [7, 8].

We have previously reported an integrated model involving Systems Biology approach, incorporating an extensive genome wide evaluation, as well as understanding the sites of mutations in 1623 genome of clinical isolates of Mtb, to identify 33 potential non-toxic metabolic targets [9, 10]. Our previous work emphasizes the use of systems biology approach to identify novel non-toxic targets with a motivation to shorten the process of drug discovery by exploiting computational methods focusing on Mtb. In order to identify drug targets with least likelihood of side effects, all 116 in silico essential genes were compared with the human genome and human microbiome at the sequence level. Of the total of 116 essential genes obtained from in silico gene knockout, 104 genes were found to have no homology to human genome sequences. In order to build a system biology approach to identify novel non-toxic target, it is desirable that all such target genes, share no homology to human genome and least homology to microbiome, to be a part of an important metabolic pathway, and to be evolutionary invariant in the clinical isolates.

In the present study, out of these potential 33 targets, 15 proteins having available crystal structures, were evaluated for the development of novel inhibitors. These targets were found to have no significant human homology. The concept of incorporating a proteome scale analysis in understanding the sites of mutations, followed by a comprehensive structure based drug design approaches [11], and digging into the wealth of experimental data to generate potential leads against these specific targets, is presented here.

With an increase in the generation of data in medicinal chemistry (both computational and synthetic), understanding of the relationships and patterns between the available data, using in silico approaches, in order to initiate a hypothesis driven drug discovery becomes imperative [12].

The published results of GlaxoSmithKline’s (GSK) large-scale high throughput screening of a library of chemical compounds against Tb were apprehended for their unique and non-redundant chemical structures. A list of total 776 compounds, out of which 426 compounds had a predicted target (based on computational studies) and 177 were potent non-cytotoxic drug sensitive Mtb H37Rv hits identified by the company, were made available [13, 14].

A detailed chemical analysis of the existing small molecule databases, as well as the evaluation of any existing lead candidates available as Mtb inhibitors in these databases was performed for the current set of targets. We evaluated our set of potential 33 targets for their existing reported GSK inhibitors. Targets were shortlisted (Table 1); based on their availability of a GSK inhibitor in the database, Protein Data Bank (PDB) structure, essentiality (experimental/in silico) and a part of Metabolic Persister Genes (MPGs). The selected 11 targets were taken up for an extensive evaluation using various in silico drug discovery tools, involving pharmacophore analysis [15, 16], molecular docking (Glide, Schrodinger and AutoDock) [17, 18] and molecular dynamics (MD) simulations [19, 20] in a few cases, using the Schrodinger suite (2015). Polypharmacological [21] studies on the above targets, with an attempt of repositioning [22] and recalibrating the old and existing drug families, are also reported here. All the targets were pre-screened using GSK open access database and OSDDChem database (http://crdd.osdd.net/osddchem/) to generate new starting leads. Herein, we report the identification of 20 lead molecules including 4 FDA approved drugs as potential candidates for the inhibition of the proposed targets in Mtb metabolism.

**Table 1:** The output and input metabolite for the shortlisted 33 each genes

Gene	Input metabolite	Output metabolite
Targets involved in nucleic acid transactions		
Purines metabolism		
*dfrA*	7,8-dihydropteroate	Tetrahydrofolate
*folB*	7,8-dihydroneopterin	6-hydroxymethyl-7,8-dihydropterin
Pyrimidines metabolism		
*pyrF*	Phosphoribosyl pyrophosphate	Phosphoribosyl amine
*Tmk*	2′-Deoxyuridine 5′ diphosphate/2′-deoxyuridine 5′-phosphate/deoxythymidine 5′-diphosphate/thymidine monophosphate	2′-Deoxyuridine 5′-diphosphate/2′ deoxyuridine 5′-phosphate/deoxythymidine 5′-diphosphate/thymidine monophosphate
Nucleotide metabolism		
*rpiB*	Ribose-5-phosphate/ribulose-5-phosphate	Ribose-5-phosphate/ribulose-5-phosphate
*Dcd*	dCTP/dUTP	dCTP/dUTP
*atpE*	ADP	ATP
*nrdI*	met-NrdF_ox_	met-NrdF_red_
DNA replication		
*nrdF2*	Ribonucleotides	Deoxyribonucleotides
RNA pseudouridine synthesis		
*Rv1711*	Pseudouridineguide snoRNAs (Pseudouridine)	RNA pseudouridine
Targets involved in membrane biosynthesis		
Fatty acid metabolism		
*fcoT*	Acyl-ACP	Fatty acids
*acpM*	FASII complex	AcpM (FAS-II complex)
*desA2*	Stearoyl-CoA (saturated fatty acids)	oleoyl-CoA (unsaturated fatty acids)
*echA3*	Δ2-enoyl-CoA	3-hydroxyacyl-CoA
*echA18.1*	Δ2-enoyl-CoA	3-hydroxyacyl-CoA
Targets involved in carbohydrate metabolism		
Kerb cycle		
Carbohydrate metabolism		
*pntAb*	Ethanol/citrate/Fd_red_^2−^	Acetyl-CoA/2-oxoglutarate/Fd_ox_
*nuoA*	NADH	NAD^+^
*canB*	CO_2_	Bicarbonate
Electron transport cycle		
*ctaE*	Cytochrome_red_	Cytochrome_ox_
*Rv0763c*	NADP^+^ reductase_ox_	Ferredoin NADP^+^ reductase_red_
*nrdH*	CDP/UDP	dCDP/dUDP
Mycothiol biosynthesis		
*Mca*	(Mycothiol (MSH)/MS-electrophiles (MSR)	AcCys + GlcN-Ins AcCySR (N-acetyl-CyS-conjugate)/(mercapturic acid) + GlcN-Ins
Targets involved in de novo pathways		
Essential cofactors		
*kdtB*	4′-phosphopantetheine	3′-desphospho-coenzyme A
*Rv2361c*	Isopentenyldiphosphate	Decaprenyldiphosphate
*Mog*	Molybdopterin	Adenylatedmolybdopterin
*moaD2*	Cyclicpyranopterin monophosphate/molybdopterin converting factor	Molybdopterin/molybdenum cofactor
Vitamin biosynthesis		
pdxH	Pyridoxamine 5′-phosphate	Pyridoxal 5′-phosphate
Amino acid biosynthesis		
*prsA*	Ribose-5-phosphate	5-phospho-α-d-ribose 1-diphosphate
*Gap*	D-glyceraldehyde 3-phosphate	3-phospho-d-glyceroyl phosphate
Peptide metabolism		
dapE	CysGly + Glu/N-succinyl-ll-2, 6-diaminoheptanedioate	Cys + Gly/succinate + ll-2,6-diaminoheptanedioate
Carbon, nitrogen and sulfur metabolism		
Rv3600c	Pantothenate	4′-phosphopantothenate

The integrated analysis reported here, includes in silico toxicity evaluation for both the targets and the molecules; involves the consideration of the drug resistance and therefore, has a potential to generate new drug candidates. These can, thus be taken up for in vitro and in vivo screening against H37Rv and MDR strains of Mtb. The study should also serve the wider anti-tuberculosis research community by providing a list of genes and their potential inhibitors that are more likely to be validated for Tb drug discovery and development.

## Methods

### Target selection and validation

All the 33 crucial metabolic genes were analyzed for their series of biochemical steps. Literature mining was used as a key source to elucidate their metabolic interference and the collected data was verified through the pathways available in KEGG, BioCyc and MetaCyc. Of these 33 targets, 15 with available crystal structures were utilized for the Molecular Docking studies. The GLIDE suite of Schrodinger for the corresponding natural substrate (NS, if any)/PDB ligand was used to determine the binding poses for each protein structure of the shortlisted 15 targets.

**Fig. 1:**
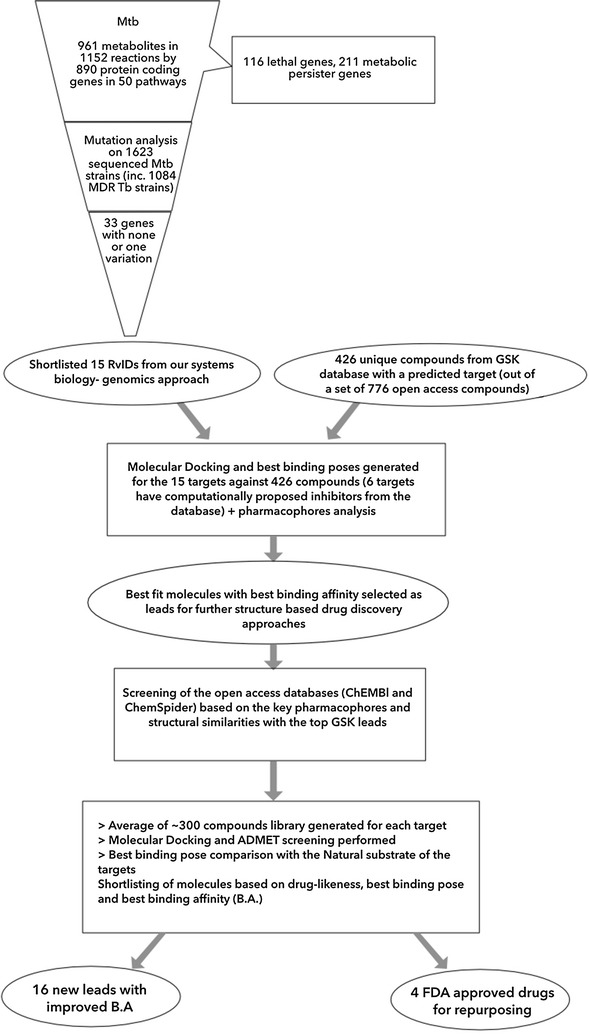
Systematic work flow explaining the methodologies and corresponding results of the analysis

Based on the understanding of the active site of the proteins, libraries of compounds (average of ~ 300) from the existing chemical databases [23] were generated for each of these targets using primarily ChEMBL database. Multi-ligand molecular docking studies were carried out for the target-specific GSK ligands/OSDDchem compounds followed by virtual screening. ADMET pharmacological properties were calculated for all the retrieved lead compounds using QikProp tool of Schrodinger. The retrieved lead compounds were further shortlisted on the basis of docking score/binding energy and no violation to Lipinski’s rule of five and other parameters. Some of these were further employed to molecular dynamic studies in order to validate the results. Schematic representation of the workflow is shown in Fig. 1.

### Softwares used for the in silico studies

Pharmacophore analysis was performed using the e-pharmacophore script and ADMET property calculations were performed using QikProp tool of Schrodinger (Small-Molecule Drug Discovery Suite 2016-3: QikProp, version 4.9, Schrödinger, LLC, New York, NY, 2016). All the compounds were initially treated with LigPrep (Schrödinger Release 2016-3: LigPrep, version 3.9, Schrödinger, LLC, New York, NY, 2016). All the implementation was carried out with the graphical user interface (GUI) of the Maestro software package (Schrödinger Release 2016-3:MacroModel, version 11.3, Schrödinger, LLC, New York, NY, 2016) using the OPLS forcefield [19, 24]. Molecular mechanic-born and surface area continuum solvation (MM/GBSA) method, to estimate the free energy of the binding of small ligands to biological macromolecules, was performed using Small-Molecule Drug Discovery Suite 2016-3: Schrödinger Suite 2016-3 QM-Polarized Ligand Docking protocol; Glide version 7.2, Schrödinger, LLC, New York, NY, 2016. MD simulations were performed using DESMOND, Schrodinger, with OPLS as the force field, TIP4P as the water model and fixing no. of Na^+^ = 7. Additionally, molecular docking on a few targets was performed using AutoDock Vina(version 1.1.2) and AutoDock tools(version 1.5.6).

The “drug-likeness” test was carried out using Lipinski’s “Rule of Five”, ro5 [25]. The distributions of the compound molecular weights (MW), (Ntie-Kang Springer Plus 2013, 2:353 Page 2 of 11 http://www.springerplus.com/content/2/1/353), lipophilicity (QlogP), number of hydrogen bond acceptors (HBA) and number of hydrogen bond donors (HBD) were calculated and used to assess the “drug-likeness” of the generated library of compounds. The 24 most relevant molecular descriptors calculated by QikProp are used to determine the #star parameter (Schrödinger 2015d). The plot of distributions of violations of Lipinski’s ro5 and Jorgensen’s ro3 within the libraries, (Ntie-Kang Springer Plus 2013, 2:353 Page 3 of 11 http://www.springerplus.com/content/2/1/353) for the respective libraries were defined as (MW < 500; log P < 5; HBD ≤ 5; HBA ≤ 10)(32), (150 ≤ MW ≤ 350; log P ≤ 4; HBD ≤ 3;HBA ≤ 6) [26, 27] and (MW ≤ 250; −2 ≤ log P ≤ 3; HBD < 3; HBA < 6; number of rotatable bonds, (NRB < 3) [28]. An example, a few selected descriptors based plots for the target Rv2763c is provided in (Additional file 1: Figures S16–S18).

The activity analysis was done based on the binding affinity score obtained either using Glide or AutoDock. The selection cut off was based on the binding affinity of the natural substrate/PDB ligand (wherever applicable) with the protein. The molecules were shortlisted based on the minimum requirement of the binding affinity/docking score, with highest number of H-bonded interactions and the best possible conformation, in alignment with the PDB ligand.

## Results

### Analysis of 33 non-toxic targets and their metabolic role

As previously stated, we short-listed 33 invariant genes in Mtb using system biology approach which are potential non-toxic candidates for drug targets. These 33 targets were classified on the basis of their nature of metabolic action viz. fatty acid metabolism or nucleotide biosynthesis. From our previous annotations [9], it was observed that of these 33 invariant genes, the functions of all the genes were known except for the two (Rv0390 and Rv1508A). Although, the function of the targets Rv0390 and Rv1508A were unknown, they were found to be essential and evolutionarily conserved. The analysis revealed that the remaining 31 genes were involved in the functioning of essential metabolic pathways without any redundant allies to replace them in their absence. The output and input metabolite for each gene has been presented in the Table 1. These target genes were classified mainly on the basis of their involvement in DNA transactions, nucleotide biosynthesis, carbohydrate metabolism and de novo pathways.

In our previous work, we reported that almost all of these 33 non-toxic targets were found to have a good Druggable Score (DS Index), and were falling in the category of highly druggable and druggable targets. It was observed that of these 33 targets, 15 had available crystal structure, which were well elucidated and were taken up for structure based drug designing.

#### Molecular docking studies of the shortlisted targets

All the 15 shortlisted targets from our previous analysis [10] were evaluated using a myriad of structure based drug design approaches. Molecular docking was performed for targets with their corresponding Natural Substrates (NS)/PDB ligand and a maximum of ten different poses were generated for each, in order to understand the best binding poses in the pocket. The targets were pre-screened against the entire 426 GSK molecules and 1192 OSDDChem database compounds to generate the initial starting leads. The selective targets were also screened with their corresponding reported GSK inhibitors, using molecular docking, to understand the binding modes. Based on the best-docked and the best-superimposed molecules with the PDB ligands, libraries of compounds were generated [from ChEMBL (https://www.ebi.ac.uk/chembl/ws) and ChemSpider (http://www.chemspider.com/)] [29] databases, having structural similarities with the leads. Pharmacophore analysis was performed for evaluating the essential pharmacophores (H-bond donors, acceptors, aromatic ring, etc.) for best binding, followed by a virtual filtering of the molecules from databases based on these essential pharmacophores. However, of these 15 selected targets (with available crystal structure), only 11 produced lead compounds (Tables 2, 3) with good docking score/binding energy and no violation to Lipinski’s rule of five and other parameters. Docking figures and interaction diagrams for the best compounds are provided in the (Additional file 1: Figures S1–S15).

**Table 2:** Shortlisted invariant RvIDs

S.no	RvIDs/gene names	Functions	Docking with corresponding NS with targets (PDB ID)	Computationally proposed GSK Compound (Docking score)	Other leads generated from 426 GSK molecules (Docking Score)
1	Rv2763c, dfrA^a,b, d^	Essential step for de novo glycine and purine	Yes (1DG5)	Total 24 inhibitors CHEMBL2098242 (Docking score = − 10.28)	SB-439950 (Docking score = − 10.88) and CHEMBL2098242 (Docking score = − 10.28)
2	Rv1094, desA2^a^	Conversion of saturated fatty acids to unsaturated fatty acids	No NS (1ZA0)	GR119270B (Docking score = − 5.09)	CHEMBL535116 (Docking score = − 6.79)
3	Rv3247c, tmk^a^	Probable Thymidylate Kinase TMK (dTMP Kinase) (Thymidilic Acid Kinase)	Yes (1G3U)	GW663013X (Docking score = − 2.60)	CHEMBL2098151 (Docking score = − 11.08)
4	Rv3607c, folB^a, c, d^	Dihydroneopterinaldolase	Yes (1NBU)	GSK2168465A (Docking score = − 4.2)	CHEMBL2097950 (Docking score = − 6.88)
5	Rv3048c, nrdF2^a^	Involved in the DNA replication pathway	No NS (1UZR)	GR119270B (Docking score = − 3.90)	CHEMBL2098385 (Docking score = − 9.01)
6	Rv2965c, kdtB	Phosphopantetheineadenylyltransferase	No NS (3PNB)	SKF-67461 (Docking score = − 3.39)	CHEMBL2097847 (Docking score = − 6.92)
7	Rv2361c, uppS^a, c^ (No GSK inhibitor reported)	Z-decaprenyl Diphosphatesynthase	Yes (2VG2)	No reported inhibitor	CHEMBL2098151 (Docking score = − 12.62)

^a^Essentiality based on experimental results

^b^Essentiality based on in silico analysis

^c^Metabolic Persister Genes

^d^MD Simulations performed

**Table 3:** Second set of shortlisted invariant RvIDs (with no GSK inhibitor)

S.no	RvIDs/gene names	Functions	Docking with corresponding NS with targets (PDB ID)	Docking with OSDDChem database (B.E. value in kcal/mol)	Other leads generated from ChEMBL database
1	Rv0865	Involved in molybdopterin biosynthesis	No natural substrate 2G4R	Compound_632, (ΔG/B.E. = − 9.9) Compound_628, (ΔG/B.E. = − 9.9)	ChEMBL255979 (B.E = − 9.9)
2	Rv0321	Interconversion of dCTP and dUTP	Yes 2QXX -TTP	Compound_633, (ΔG/B.E = − 9.9) Compound_410, (ΔG/B.E = − 9.9) Compound_105, (ΔG/B.E = − 9.9)	ChEMBL533912 (B.E = − 9.3)
3	Rv0098	Involved in fatty acid metabolism	Yes 2PFC-PLM	Compound_1029, (ΔG/B.E = − 9.1) Compound_1030, (ΔG/B.E = − 9.1)	ChEMBL3349754 (B.E = − 8.6) ChEMBL3037996 (B.E = − 9.1)
4	Rv0390	Function unknown Rhodanese-related sulfurtransferase	No natural substrate 2FSX	Compound_14, (ΔG/B.E = − 9.9) Compound_13, (ΔG/B.E = − 9.9)	ChEMBL217735 (B.E = − 8.0) ChEMBL76817 (B.E = − 8.0)
5	Rv3588c	Catalyzes reversible dehydration of CO2 to form bicarbonate			No leads
6	Rv2244^a^	Involved in fatty acid biosynthesis (mycolic acids synthesis)	1KLP (Solution Structure)		No leads
7	Rv2465c	Interconverts ribose-5-phosphate and ribulose-5-phosphate	2VVP	SDF_file14_out − 9.9	No leads
8	Rv2607	Involved in biosynthesis of pyridoxine (vitamin B6) and pyridoxal phosphate	2A2J	All the 1192 compounds were used and none of the OSDDChem compounds were docked to the corresponding target	No leads

Screening performed with the OSDDChem database

^a^Essentiality based on experimental results

To predict the drug-likeness and pharmacological properties of the compounds, various descriptors were calculated, theoretically. Some of these descriptors were plotted against the compound numbers, for graphical representations (example shown for Rv2763c in the (Additional file 1: Figures S16–S18). Based on these analyses, we generated a list of compounds (Table 4) as potential inhibitors. These potential candidates should, ideally, provide a better efficacy as compared to the current set of drug candidates. This evaluation helped us in picking up compounds from open access databases, which can be procured readily and can be taken up further for in vitro and in vivo analysis. The potential 20 lead compounds proposed here also include 4 known FDA approved drugs (Listed in Table 4), which could be utilized for repurposing, in combination with the current regime, for a speedy drug discovery process.

**Table 4:** Lead molecules identified based on the best docking scores, binding affinity calculations, and best superimposition with the natural substrate

Target	Compound name	Structure	Docking score/B.E.
Rv2763c	CHEMBL432987		Docking score = − 12.08
Rv2763c	CHEMBL2098242		Docking score = − 10.28
Rv2763c	CHEMBL32039 (Tetroxoprim)		Docking score = − 10.19
Rv3607c	CSID:20211002		Docking score = − 7.41
Rv3247c	CHEMBL3184131		Dockingscore = − 11.55
Rv3247c	CHEMBL1467435		Docking score = − 11.32
Rv3247c	CHEMBL20734 (Nemonapride)		Docking score = − 10.67
Rv3247c	ChEMBL219916 (Domperidone)		Docking score = − 9.17
Rv0321	ChEMBL533912		B.E. = − 9.3
Rv3048c	CHEMBL2098385 (From GSK open access compounds)		Docking score = − 9.01
Rv3048c	CSID:353848		Docking score = − 7.41
Rv0098	ChEMBL3037996		B.E. = − 9.1
Rv0390	ChEMBL217735		B.E. = − 8.0
Rv0098	ChEMBL3349754		B.E. = − 8.6
Rv1094	CHEMBL535116		Docking score = − 6.79
Rv1094	CHEMBL3302699 (Droxidopa)		Docking score = − 6.68
Rv2965c	CHEMBL2097847		Docking score = − 6.92
Rv2361c	CHEMBL2098151 (From the set of GSK molecules)		Docking score = − 12.62
Rv0865	ChEMBL255979		B.E. = − 9.9
Rv0390	ChEMBL76817		B.E. = − 8.0

As a part of this analysis, we also observed that the binding pocket in dfrA (Rv2763c) for Trimethoprim has a single point mutation (A29T), in only one of the MDR strains. It is proposed that the emerging mutations could result in the development of resistance against trimethoprim [30] and hence would eventually require better alternatives. We found another FDA approved drug, Tetroxoprim (Table 4), showing an improved binding affinity with the target, in the same binding pocket as Trimethoprim. This can thus, be proposed as an alternative to the drug Trimethoprim, for the combination therapy against Tb.

## Discussion

We have thoroughly investigated the 15 metabolic genes (in silico and experimentally essential genes as well as a metabolic persister gene), in Mtb, which are highly invariant across the available 1623 strains including 1084 MDR strains of the bacteria, for detailed structure based drug discovery approaches. The Mtb specific invariant genes in the available genome were evaluated for their relevance in drug discovery, as these genes can form good targets for the inhibition of the growth of the organism. Based on the metabolic pathway analysis, it was observed that all of these 15 genes were found to be crucial candidates for structure based drug designing and none of the gene showed any convergence. The genes were found to act on the specific input metabolite, thereby suggesting that these metabolites can be further exploited to discover drugs based on the specific essential metabolic pathways. The analysis of input and output metabolites for the short-listed 15 genes revealed that all the genes, except Rv0390 (with unknown function), are involved in specific functions, without any interference amongst their primary metabolites, in any of their metabolic pathways. As there was no interference in the metabolic pathways, all the genes were considered as independent structure specific drug targets. This makes every gene unique in its action and thereby suggested that if a drug is designed against these essential genes, it will remain highly specific in the inhibition of metabolic pathway of Mtb by effectively acting on them. The absence of any convergence in the mechanistic action of these genes ensured that the functioning of the drug will not bring about any other stochastic damage and will be highly exclusive in its action. The enhanced functional annotations of the Mtb genome, obtained through a crowd sourcing approach was previously used by us to reconstruct the metabolic network of Mtb in a bottom up manner [9]. It is understood that the possible limitation of assuming pathway independence lies in the extent to which all the pathways and their interconnections are reported in literature. However, given that literature might not be comprehensive and every interconnection between pathways might not be known, there exist a slight possibility of these shortlisted genes ending up in same unique pathway. With the well-characterized PDB data, these genes were analyzed and subjected to conformational analysis for structure dependent drug designing.

### A) Targets involved in DNA transactions

#### Purines metabolism

##### 1) Rv2763c (dfrA/folA)

The gene is involved in an essential step in de novo glycine and purine synthesis and dihydrofolate reductase activity. In folate biosynthesis, dihydrofolate reductase coded by dfrA catalyses the reduction of folate to 5, 6, 7, 8-tetrahydrofolate. Molecular docking was carried out on a set of reported 24 GSK inhibitors (for folA) and it was found that SB-439950 in the NAD binding pocket and ChEMBL2098242 in the Trimethoprim binding pocket exhibited the docking score of − 10.88 and − 10.28 respectively (Table 2). Structural and pharmacophore similarities with NS and the GSK inhibitor (Additional file 1: Figures S1, S2), resulted into a set of 830 molecules, where ChEMBL432987 showed a highest docking score of − 12.085 and ChEMBL32039 exhibited a docking score of − 10.19 (Table 4). The interaction analysis of ChEMBl2098242 revealed that NH_2_ and NH are involved in the hydrogen bonding with Asp27, Ile94, and a Phe31 Pi-stacking.

##### 2) Rv3607c (folB)

The gene is a MPG, which is experimentally essential and is involved in dihydroneopterin/folate biosynthesis. Binding studies were carried out in reference to the NS to understand the poses and interactions. Molecular docking was performed for all the GSK molecules including the reported GSK inhibitor (GSK2168465A; docking score = − 4.21) (Table 2). A compound library of ~ 1200 compounds was generated and evaluated using molecular docking studies (CSID: 20211002; best docking score = − 7.41) (Additional file 1: Figure S3) (Table 4).

#### Pyrimidines metabolism

##### 3) Rv3247c (tmk)

The gene is a thymidylate kinase (dTMP Kinase). molecular docking was carried out with all the GSK molecules as well as the proposed inhibitors (docking score = − 2.6) (Table 2). A compound library of 450 compounds was generated with high structural similarities with the best GSK molecules (Additional file 1: Figures S4, S5).

On analysis, it was observed that four lead compounds ChEMBL3184131, ChEMBL1467435, ChEMBL20734 and ChEMBL219916 exhibited the strong binding affinity with the docking score of − 11.55, − 11.32, − 10.67 and − 9.17 respectively (Table 4).

#### Nucleotide metabolism

##### 4) Rv0321 (dcd)

The gene is involved in the interconversion of dCTP and dUTP and did not have a reported GSK inhibitor. Therefore, OSDDChem database was screened against the target to identify the top 100 compounds exhibiting highest binding energy, better than the NS (docking score = − 9.9).Clustering was carried out for the top ranked compounds, leading to the generation of a pharmacophore model, with survival score of 3.43 (Additional file 1: Figure S6a). In order to validate the quality of the generated pharmacophore model, clinically approved Tb drug Rifampicin showed a two-feature mapping with good fit value of 4.74.A molecular library (~ 1000 compounds) was generated using various databases, based on the best structural and pharmacophore similarities. The best binding affinity was obtained for ChEMBL533912 with ΔG score of − 9.3 kcal/mol (Table 4). The lead compound showed hydrogen bond interactions between NH of propanamide flanked in the flurophenyl with Tyr162. Nitrogen atom in the 1, 2, 4 triazol ring showed interactions with Ala167 and Ser161 with an interatomic distance of 3.5 Å each respectively.

#### DNA replication

##### 5) Rv3048c (nrdF2)

The gene is involved in the DNA replication pathway. It has no NS attached in its PDB structure. Molecular docking studies were performed with the reported GSK molecules and the entire GSK set of molecules for comparison (Table 2). Library of compounds (~ 350 molecules) was generated based on structural and pharmacophore similarities. ChEMBL2098385 and CSID353848 exhibited a highest binding affinity and the best docking score of − 9.01 and − 7.41 respectively (Additional file 1: Figures S7, S8) (Table 4).

### B) Targets involved in membrane biosynthesis

#### Fatty acid metabolism

##### 6) Rv0098 (fcoT)

The gene is a long chain acyl-coenzyme A (CoA) thioesterase that hydrolyses fatty acyl-CoA to fatty acid, hence involved in fatty acid metabolism. Top 100 compounds (with improved binding energy as compared to the NS, ΔG score of − 6.9 kcal/mol), were identified by virtual screening of the OSDDChem database. Library of ~ 1000 molecules was generated based on the structural and pharmacophore similarities. This library was further screened against the target. Two lead molecules, ChEMBL3349754 and ChEMBL3037996 exhibited binding affinity ofΔG = − 8.6 and − 9.1 kcal/mol, respectively (Table 4) (Additional file 1: Figure S9).

The interaction study of ChEMBL3349754 revealed that the carbonyl group of the phenyl acetate ring showed interactions with Asn83 which is also present in the binding site with an inter atomic distance of 3.1 Å and oxygen atom present in the eleventh position of trioxatricyclo rings showed strong interactions with Leu115 and tyr87 present in the binding site at a distance of 3.4 and 3.4 Å.

##### 7) Rv1094 (desA2)

The gene is involved in conversion of saturated fatty acids to unsaturated fatty acids. In the biosynthesis of unsaturated fatty acids, the gene codes for acyl-[acyl-carrier-protein] desaturase which catalyses the conversion of stearoyl-CoA to oleoyl-CoA. It has no NS reported in its PDB structure. All the GSK molecules and reported GSK inhibitors were screened with the protein, in the binding pocket generated using SiteMap tool of Schrodinger. A compound library (180 compounds) was screened against the target. ADMET property prediction (QikProp, Schrodinger) and the docking studies with the known drug molecules (based on structural similarities, generated using QikProp) were also carried out (Additional file 1: Figure S10). ChEMBL3302699 exhibited a docking score of − 6.68 whereas ChEMBL535116 showed the strong binding affinity of − 6.79 with the existing drug Droxidopa, which is a synthetic amino acid precursor and acts as a prodrug to the neurotransmitter norepinephrine (Table 4). ChEMBL535116 showed hydrogen bond interactions with Trp32 and Glu29 and a Pi-stacking with Trp32 and Arg102.

#### Targets involved in de novo pathways (Essential cofactors)

##### 8) Rv2965c (kdtB)

The gene is involved in CoA biosynthesis (4th step) and reversibly transfers an adenylyl group from ATP to 4′-phosphopantetheine, yielding dephospho-CoA (DPCOA) and pyrophosphate. There is no NS attached to its PDB structure, however it has a CoA. Receptor grid was generated using this CoA and SiteMap (Schrodinger) predictions of the binding pocket. Molecular docking was carried out with the reported GSK molecules as well as the entire GSK library to compare the results (Table 2). Compound library (~ 50) was generated using similar structural analysis of the GSK molecules (Additional file 1: Figure S11). ChEMBL2097847 exhibited a docking score of − 6.92 (Table 4).

##### 9) Rv2361c (uppS)

The gene is involved in Z-decaprenyldiphosphate synthesis. The gene codes for a protein, which is involved in the synthesis of decaprenyldiphosphate, a molecule with a critical role in the biosynthesis of most features of the mycobacterial cell wall. The gene is also a part of MPGs. Molecular docking was performed with NS and top 10 poses were generated (Additional file 1: Figure S12). A library of molecules (~ 800 compounds) was generated based on the best binding from the set of 426 GSK molecules (Table 2). Highest docking score achieved for the compound ChEMBL2098151was − 12.62 (Table 4). The compound showed most important interactions of Arg244, Ser252, Arg292 and Arg250 with the cyclopropyl ester functionality. The interaction analysis also revealed an important Pi- interaction, which results in a drastic increase in the binding of the pyridine ring with Arg127.

##### 10) Rv0865 (mog)

The gene is associated with the molybdopterin biosynthesis in Mtb. It has no NS/PDB ligand associated with the crystal structure. The OSDDChem library was computationally screened against the binding pockets of the target protein using AutoDock Vina. Molecular docking carried out on a set of 100top scored pose ligands exhibited strong binding affinity (ΔG value between − 8.5 and − 9.9 kcal/mol) and were further selected for compound clustering. The cluster generated from fingerprint based similarity and chemical clustering was used for the development of feature models. Pharmacophores were derived for the clustered and structurally similar compounds (matching to the feature model) available in ChEMBL and ChemSpider databases. Pharmacophores satisfying drug-like properties were further employed for virtual screening. Highest binding free energy obtained for ChEMBL255979 was ΔG as − 9.9 kcal/mol (Table 4). Molecular binding interaction of the protein complex revealed that carboxyl group which is placed in-between trimethyldecahydro-3, 12-epoxy and biphenyl ring showed interactions with Val11 at an atomic distance of 3.6 Å and the same carboxyl group showed two hydrogen bond interactions with Ser13 with a bond length of 3.1 and 3.3 Å respectively (Additional file 1: Figure S6b).

#### Targets with unknown function

##### Rv0390

This is a gene with undefined function. A diverse set of OSDDChem database, containing 1192 compounds, was docked and a series of top scoring compounds with ΔG = − 6.8 kcal/mol or above, were obtained. Clustering analysis was performed to determine the structural similarity between compounds. The large cluster representative structures were employed for the development of pharmacophore models, and compounds with survival score of 3.54 were considered to be active in the set. 3-dimensional Pharmacophore based virtual screening resulted in the retrieval of top ranked 100 compounds. Of these, two lead compounds viz., ChEMBL217735 and ChEMBL76817 exhibited the predicted binding energy of ΔG = − 8.0 kcal/mol each with acceptable pharmacokinetics properties (Table 4, Additional file 1: Figure S13). The Oxygen of butanoate moiety of ChEMBL217735 showed interactions with Ile 65 and Asp 62 at distance of 3.1 and 3.6 Å respectively. Hydrogen bond interactions were observed between carboxylate group of Ala 66 with a bond length of 3.1 Å.

Assays for the in vitro activity of dihydrofolatereductase (dfrA/folA, Rv2763c), dihydroneoterinaldolase (folB, Rv3607c), thymidylate kinase (tmk, Rv3247c) and Z-decaprenyldiphosphate synthase (uppS, Rv2361c), with the set of inhibitors having good IC_50_ and MIC-_50_ values have been reported in the literature [30–34]. We have evaluated the structural similarities of these inhibitors (reporting highest activity) with the inhibitors of the targets shortlisted in the present study. The shortlisted inhibitors developed primarily in silico were subjected to molecular docking analysis with their respective targets for comparative studies. Our studies revealed that the inhibitors proposed for targets 1G3U (tmk, Rv3247c) and 1DG5 (dfrA/folA, Rv2763c) showed better in silico binding affinity as compared to their previously reported activities using in vitro analysis. The docking score of the theoretically proposed leads for tmk 1G3U (Rv3247c, docking score = − 7.01) and folA, 1DG5 (Rv2763c, docking score = − 9.48) were found to be higher than the inhibitors with reported IC_50_ in vivo values. It may be noted that many successful inhibitors do not show the desired in vivo activity and similarly many in silico best inhibitors may not show the similar activity. However, in silico work does have a potential of reducing the failure rates and increases the chance of success in drug discovery.

As previously reported, these 15 shortlisted targets were further subjected to ‘druggability’ assessment. On analysis it was observed that out of these, 5 had unique crystal structures and 10 had multiple crystal structures available in PDB. The targets with more than 1 crystal structure were subjected to multiple sequence alignment for the selection of the best structure to be utilized for molecular docking studies. In the process, it was observed that these targets showed a significant deviation in the DS index. This suggested that the quality of the sequences of the PDB structures to be taken up for molecular docking studies play a vital role for the validity of results in a computational based study. On comparing the DS index of targets with unique crystal structures, it was observed that the ones with maximum sequence coverage exhibited high DS index as compared to the structures with minimal sequence coverage thus validating our approach for selection of potential targets, which are evolutionarily conserved as well. Therefore, this system analysis demands that the PDB structures for carrying out the analysis are relevant, only if the target sequence matches the invariant sequence of the genomes.

We had also reported the possibility of targeting NDH-I with an existing FDA approved drug for type-II diabetes, Metformin, as an adjunct therapy for Tb. Based on our previous analysis, it was evaluated that NDH-I has a putative role in giving rise to bacterial persistence [35]. Additionally, similarity searches using QikProp tool of Schrodinger yielded some existing drugs having high structural similarities with the docked molecules. As an example, the structural comparison of the best-docked molecules for target 1G3U (Rv3247c), revealed Domperidone and Nemonapride (selective antagonist of the dopamine D_2_ and D_3_ receptors), as probable drug candidates for repurposing. For the target 1DG5 (Rv2763c), similarity studies with the best-docked molecule showed Tetroxoprim (a less used antimalarial and a derivative of Trimethoprim), as the closest known drug, which can be taken up for repurposing (docking score = − 10.19). Along with this, Droxidopa (analog of l-Dopa) has shown a potential inhibition property for 1ZA0 (Rv1094) (docking score = − 6.68) (Table 4). We also performed an analysis in order to understand the effect of protein folding and conformational changes on the binding affinity. As an example, Rv2763c (dihydrofolate reductase) and its best PDB structure (PDB ID: 1DG5) was evaluated for comparison with its human homolog (PDB ID: 4QHV). The two proteins have very little homology in the sequence, but the structural comparisons indicated that the two proteins fold in a similar fashion. We observed that the ligand CHEMBL432987, which is the best binding molecule (docking score = − 12.08), does not bind well with the human homolog (docking score = − 8.00). This is considered as a drastic drop in the binding affinity between the two proteins. This could be attributed to the differences in the environment of both NADP and of the inhibitor between the Mtb and human structures. Residues like Ala101 and Leu102 nearing the N6 of NADP are very distinctly hydrophobic in pathogen as compared to the host [36]. It, therefore, becomes important to address that the sequence homology is not 100% indicative of the similarities in the binding sites and hence, we do need to incorporate structural comparisons (protein folding) to understand the homology between the two structures.

## Conclusion

We therefore, propose that with these methodologies, new potential drug-like leads can be generated with the success rate of 1/10 as compared to the existing 1/100 molecule entering clinical trials. These studies are expected to lead to the generation of a new anti-Tb drug candidate, primarily developed in silico. Therefore, our attempt to develop a comprehensive approach for the drug discovery by short-circuiting the research on generation of newer chemical scaffolds will positively influence the probability of clinical success of a drug candidate. We, therefore, suggest an integrated methodology, which will not only tackle the MDR form of Mtb but also the most important persister population of the bacterium.

## Additional file

**Additional file 1: Figure S1.** Docking with the best GSK molecule (docking score= −10.88) for folA (Rv2763c). **Figure S2.** E-pharmacophore of Trimethoprim (NS) Vs the library of compounds generated based on similar results forfolA (Rv2763c). **Figure S3.** Docking with NS and the best- docked molecule (docking score= −7.08) for folB (Rv3607c). **Figure S4.** Highest docking score with best binding pose match= −11.55 for tmk(Rv3247c). **Figure S5.** Top 2 poses for the best docked GSK molecule, docking score= −11.08 for tmk(Rv3247c). **Figure S6.** (a) Docking pattern of the lead compound ChEMBL533912 in to the enzyme BifunctionaldCTPdeaminase (Rv0321, PDB ID: 2QXX).ChEMBL533912 showed polar contacts with Tyr162, Ser167 and Ala167 along with NSThymidine-5’-Triphosphate –TTPrespectively.Hydrogen bond interactions are represented in yellow dotted lines. The Natural substrate is represented as red stick;(b) Docked conformation of protein Involved in Molybdopterin biosynthesis (Rv0865, PDB ID 2G4R) with top ranked ligand ChEMBL255979, showing the interaction with the crucial residues Ser13 with two hydrogen bond interactions and Val 11 in the active site. **Figure S7.** Best pose and interaction diagram for the best-docked non-GSK molecule (docking score= −7.41) for nrdF2 (Rv3048c). **Figure S8.** 2 Best poses with highest binding GSK compound and its interaction diagram (Docking score= −9.01) for nrdF2 (Rv3048c). **Figure S9.** Binding pattern of two lead compounds on to the hypothetical protein (Rv0098, PDB ID 2PFC); (**a)** ChEMBL3349754, showing the hydrophobic interaction with Asn83, Met118, Lue115 and Tyr87; (**F)**ChEMBL3037996Showed hydrophobic environment around binding site residues Met 118, Ile120 and Tyr87.Red color stick showing the NS Palmitic acid –PLM and hydrogen bond interactions are represented in yellow dotted lines. **Figure S10.** Best Docking score= −6.79 and interaction diagram obtained from the entire library of molecules for desA2 (Rv1094). **Figure S11.** Best poses for the top ranked GSK molecule for kdtB (Rv2965c). **Figure S12.** (a). Top poses of the best-docked GSK molecules, (b). Docking performed with 426 GSK molecules, (c). Interaction diagram for the best GSK molecule foruppS (Rv2361c). **Figure S13.** Docked complex of lead compounds into protein Rhodanese-related sulfurtransferase(Rv0390, PDB ID 2FSX);** (a)** ChEMBL217735Showed hydrophobic interaction with Phe64,His60,Ala66,Ilu65 and Asp62 represented by yellow dotted lines; (**b)** ChEMBL76817Showed hydrophobic interaction with binding site residues Arg 71are represented by yellow dotted lines. **Figure S14.** Generated Common Pharmacophore hypotheses for a set of 5 targets as: (a) ARR : (b) AAP, (c) AHR: (d) AAP: (e) AAH (green sphere/circle: hydrophobic group, orange ring: aromatic ring, Pink sphere/circle: hydrogen bond acceptor, light-blue sphere: hydrogen bond donors, blue sphere positively charged group). **Figure S15.** Pharmacophore feature(AHR hypothesis) mapping of external test set compounds Hydrophobic features (H7) mapped over carboxyl group, and the second hydrogen bond acceptor (A2), feature mapped over alkene group of Rifampicin with fit value of 4.74. **Figure S16.** Distributions of violations of Lipinski’s ro5 and Jorgensen’s ro3 within the compound library for Rv2763c. **Figure S17.** Scatter diagrams showing pair wise distribution of “drug-likeness” descriptors, MW against Predicted Octanol-Water Coefficient,QlogP for Rv2763c. **Figure S18.** Histogram plot of the count of the compounds VsMol. Wt. and Predicted Octanol-Water Coefficient, QlogP, Drug like range: −2.0–6.5, for Rv2763c.

## Abbreviations

Tb: tuberculosis
Mtb: *Mycobacterium tuberculosis*
SDGs: sustainable development goals
MDR: multi drug resistant
XDR: extreme drug resistant
OSDD: open source drug discovery
GSK: GlaxoSmithKline
PDB: protein data bank
MPGs: metabolic persister genes
MD: molecular dynamics
DS: druggable score
NS: natural substrates
ADMET: absorption, distribution, metabolism, excretion, toxicity
GUI: graphical user interface
MW: molecular weights
HBA: hydrogen bond acceptor
HBD: hydrogen bond donor
NRB: number of rotatable bonds
