# Supplementary material for: Structure based drug discovery for designing leads for the non-toxic metabolic targets in multi drug resistant Mycobacterium tuberculosis
Source: J Transl Med. 2017 Dec 21;15:261. doi: 10.1186/s12967-017-1363-9 (PMC5740895; doi:10.1186/s12967-017-1363-9)
Supplement: Supplementary file 1 — Additional file 1: Figure S1. Docking with the best GSK molecule (docking score= −10.88) for folA (Rv2763c). Figure S2. E-pharmacophore of Trimethoprim (NS) Vs the library of compounds generated based on similar results forfolA (Rv2763c). Figure S3. Docking with NS and the best- docked molecule (docking score= −7.08) for folB (Rv3607c). Figure S4. Highest docking score with best binding pose match= −11.55 for tmk(Rv3247c). Figure S5. Top 2 poses for the best docked GSK molecule, docking score= −11.08 for tmk(Rv3247c). Figure S6. (a) Docking pattern of the lead compound ChEMBL533912 in to the enzyme BifunctionaldCTPdeaminase (Rv0321, PDB ID: 2QXX).ChEMBL533912 showed polar contacts with Tyr162, Ser167 and Ala167 along with NSThymidine-5’-Triphosphate –TTPrespectively.Hydrogen bond interactions are represented in yellow dotted lines. The Natural substrate is represented as red stick;(b) Docked conformation of protein Involved in Molybdopterin biosynthesis (Rv0865, PDB ID 2G4R) with top ranked ligand ChEMBL255979, showing the interaction with the crucial residues Ser13 with two hydrogen bond interactions and Val 11 in the active site. Figure S7. Best pose and interaction diagram for the best-docked non-GSK molecule (docking score= −7.41) for nrdF2 (Rv3048c). Figure S8. 2 Best poses with highest binding GSK compound and its interaction diagram (Docking score= −9.01) for nrdF2 (Rv3048c). Figure S9. Binding pattern of two lead compounds on to the hypothetical protein (Rv0098, PDB ID 2PFC); (a) ChEMBL3349754, showing the hydrophobic interaction with Asn83, Met118, Lue115 and Tyr87; (F)ChEMBL3037996Showed hydrophobic environment around binding site residues Met 118, Ile120 and Tyr87.Red color stick showing the NS Palmitic acid –PLM and hydrogen bond interactions are represented in yellow dotted lines. Figure S10. Best Docking score= −6.79 and interaction diagram obtained from the entire library of molecules for desA2 (Rv1094). Figure S11. Best poses for the top ranked GSK m [file 12967_2017_1363_MOESM1_ESM.docx]

**Structure based drug discovery for designing leads for the non-toxic metabolic targets in multi drug resistant *Mycobacterium Tuberculosis***

Divneet Kaur^1^,Shalu Mathew^2^, Chinchu G S Nair^2^, Azitha Begum^2^,

Ashwin K. Jainanarayan^1,†^, Mukta Sharma^1^& Samir K. Brahmachari^1,2,3,4^*

^1^CSIR- Institute of Genomics and Integrative Biology, New Delhi, India

^2^Centre for Open Innovation- Indian Centre for Social Transformation, Bengaluru, Karnataka

^3^Academy of Scientific and Innovative Research, New Delhi, India

^4^CSIR- Open Source Drug Discovery Unit, New Delhi, India

**Additional Information:**


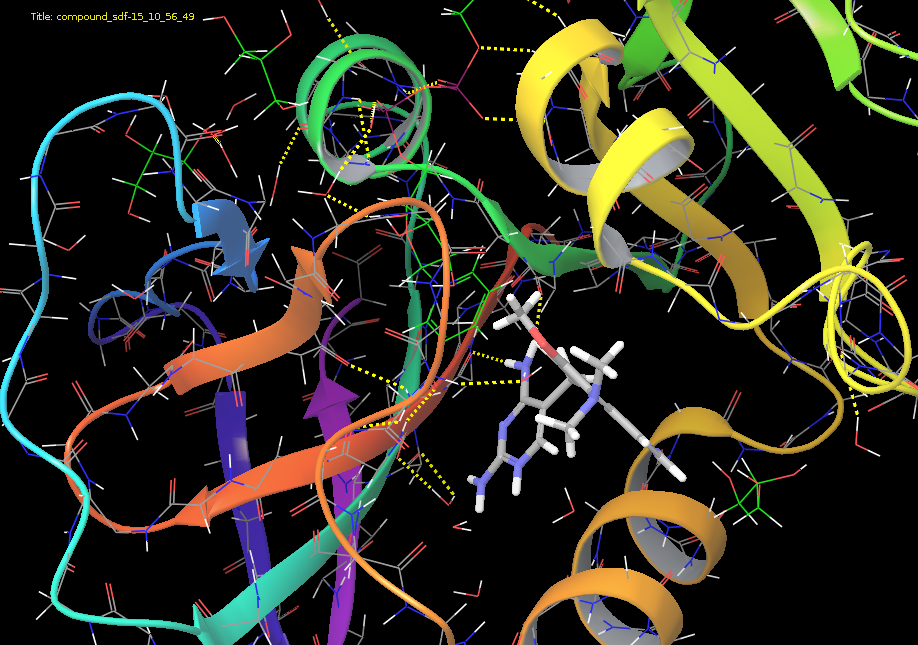


**Figure S1**: Docking with the best GSK molecule (docking score= -10.88) for folA (Rv2763c)


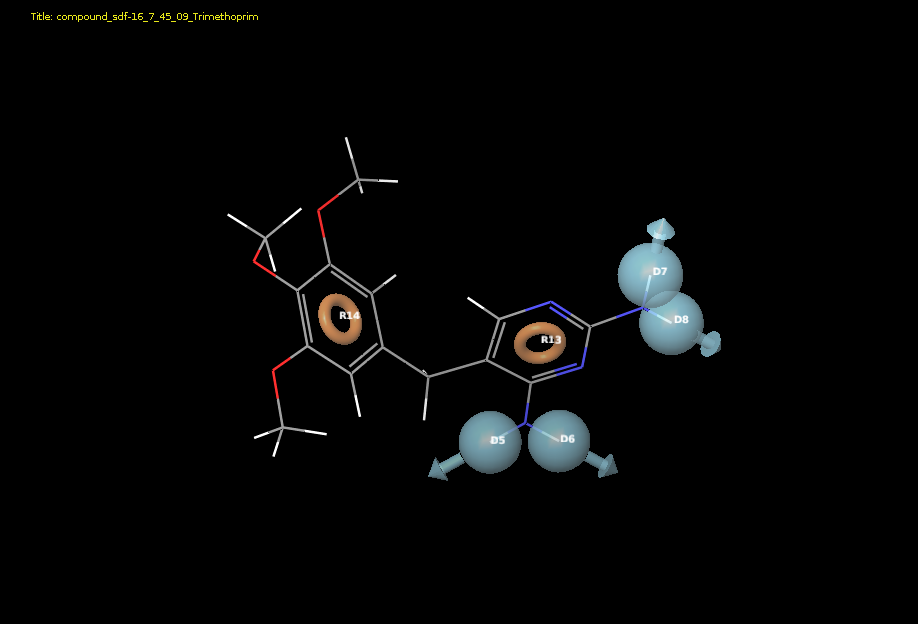

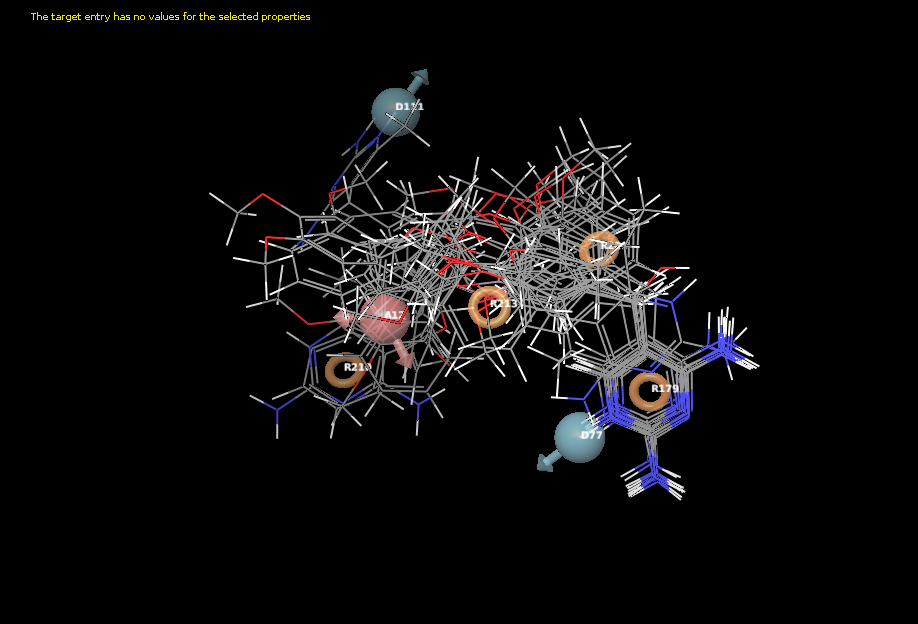


**Figure S2**: E-pharmacophore of Trimethoprim (NS) Vs the library of compounds generated based on similar results forfolA (Rv2763c)

† Presently a 3^rd^ year integrated BS-MS student of Indian Institute of Science Education and Research (IISER), Mohali, India.


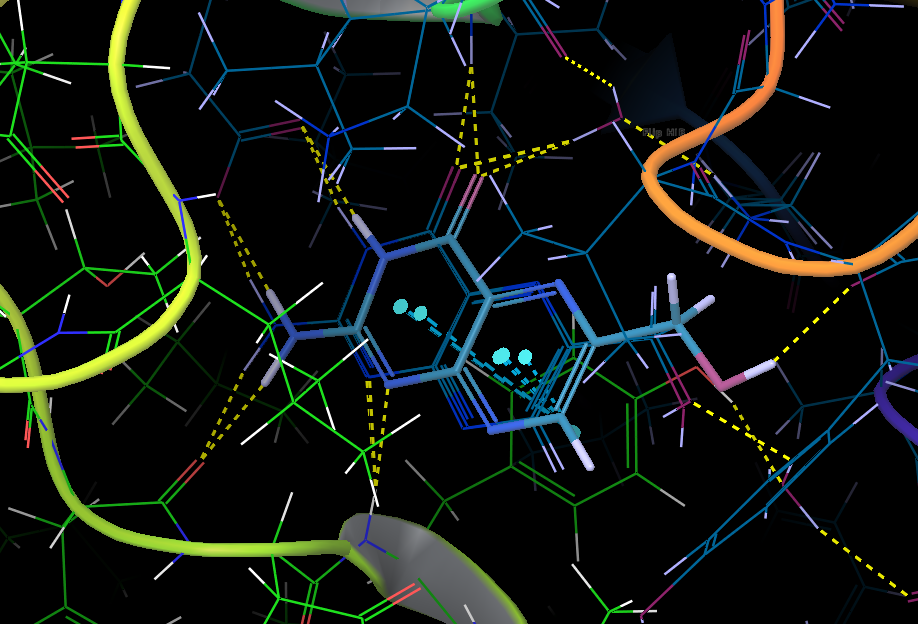

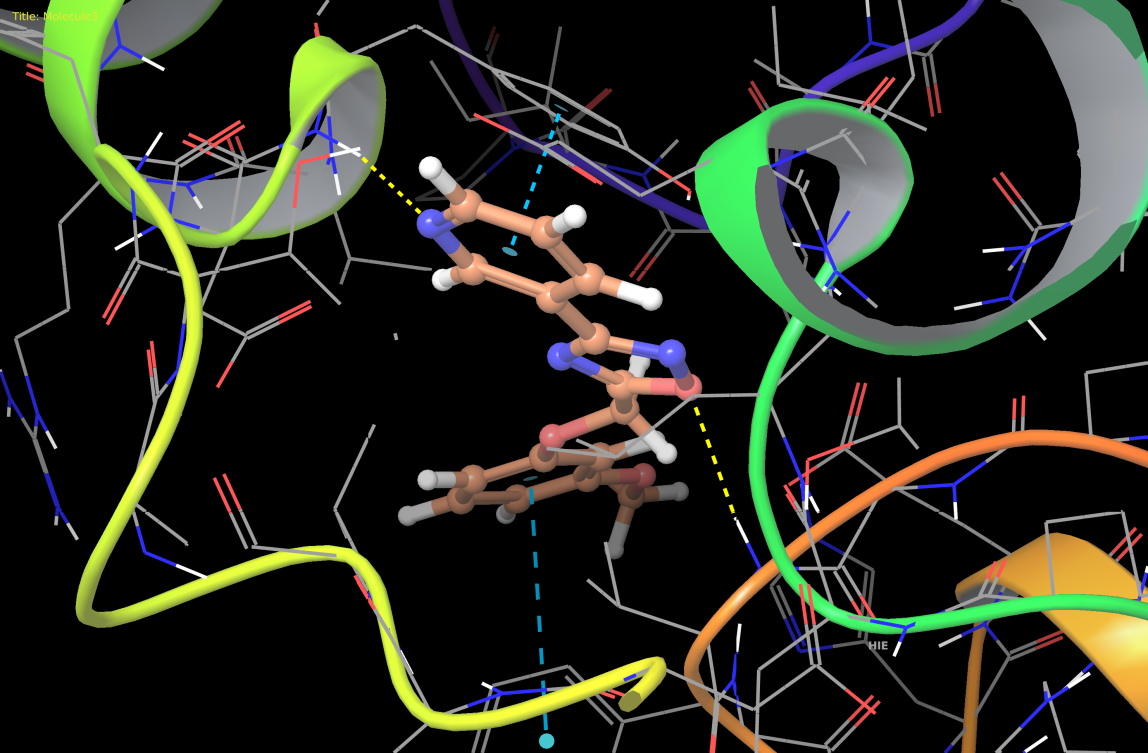


**Figure S3**: Docking with NS and the best- docked molecule (docking score= -7.08) for folB (Rv3607c).


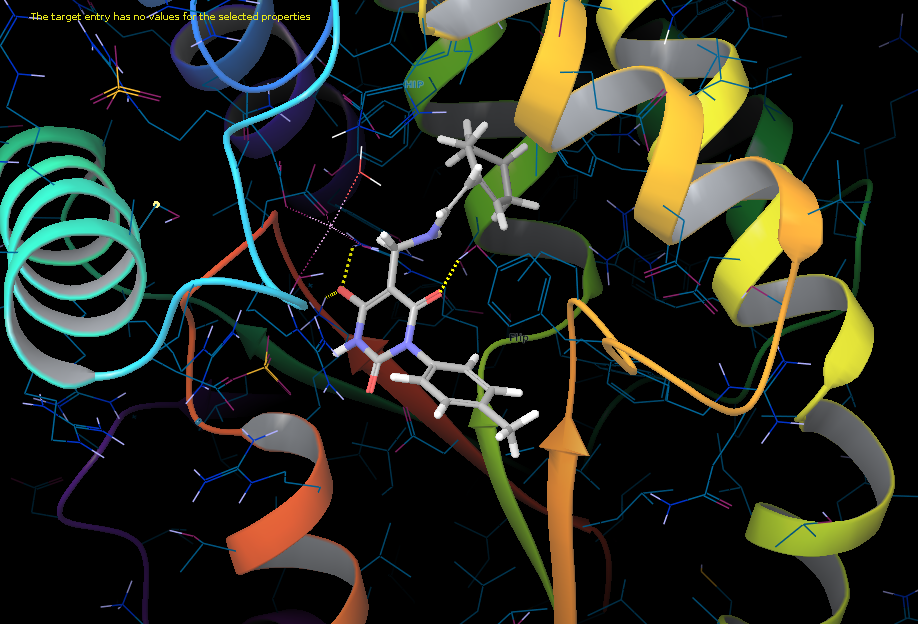

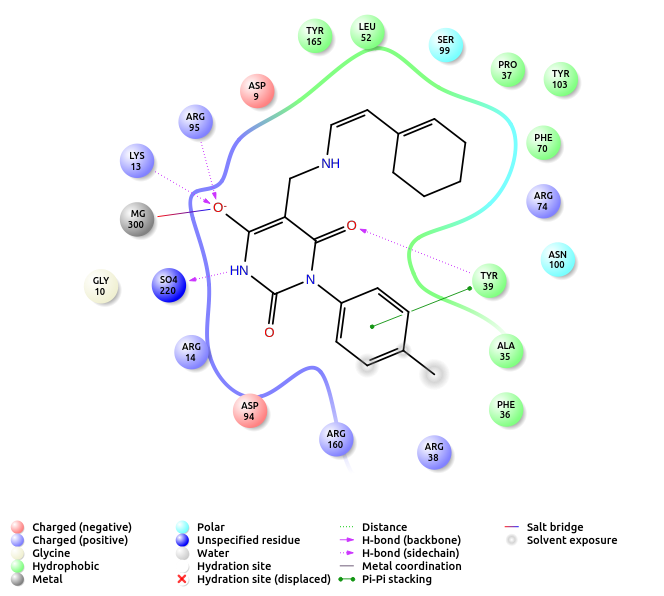


**Figure S4**: Highest docking score with best binding pose match= -11.55 for tmk(Rv3247c)


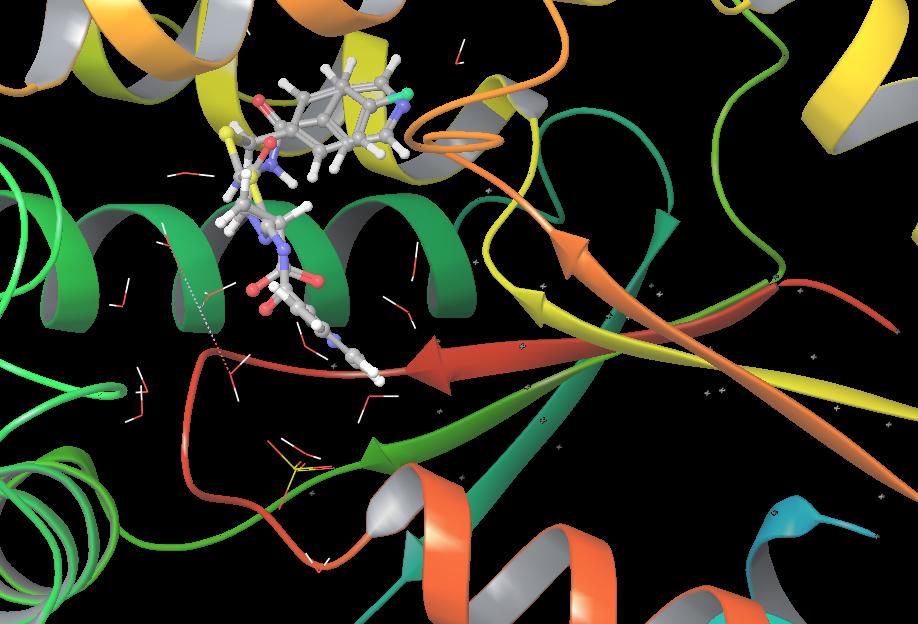


**Figure S5**: Top 2 poses for the best docked GSK molecule, docking score= -11.08 for tmk(Rv3247c)


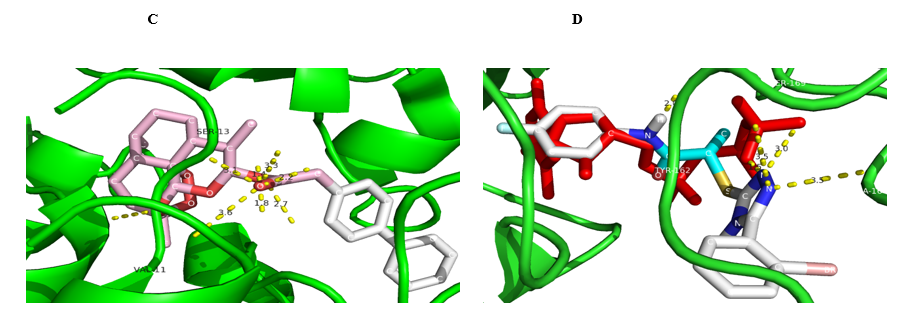
b
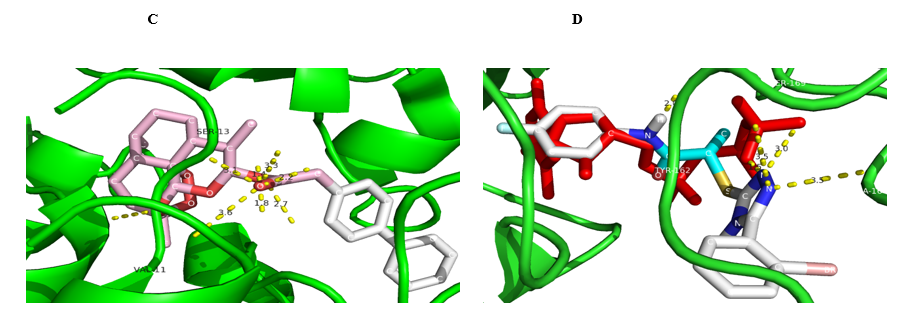


**Figure S6:**(a) Docking pattern of the lead compound ChEMBL533912 in to the enzyme BifunctionaldCTPdeaminase (Rv0321, PDB ID: 2QXX).ChEMBL533912 showed polar contacts with Tyr162, Ser167 and Ala167 along with NSThymidine-5'-Triphosphate –TTPrespectively.Hydrogen bond interactions are represented in yellow dotted lines. The Natural substrate is represented as red stick;(b) Docked conformation of protein Involved in Molybdopterin biosynthesis (Rv0865, PDB ID 2G4R) with top ranked ligand ChEMBL255979, showing the interaction with the crucial residues Ser13 with two hydrogen bond interactions and Val 11 in the active site.


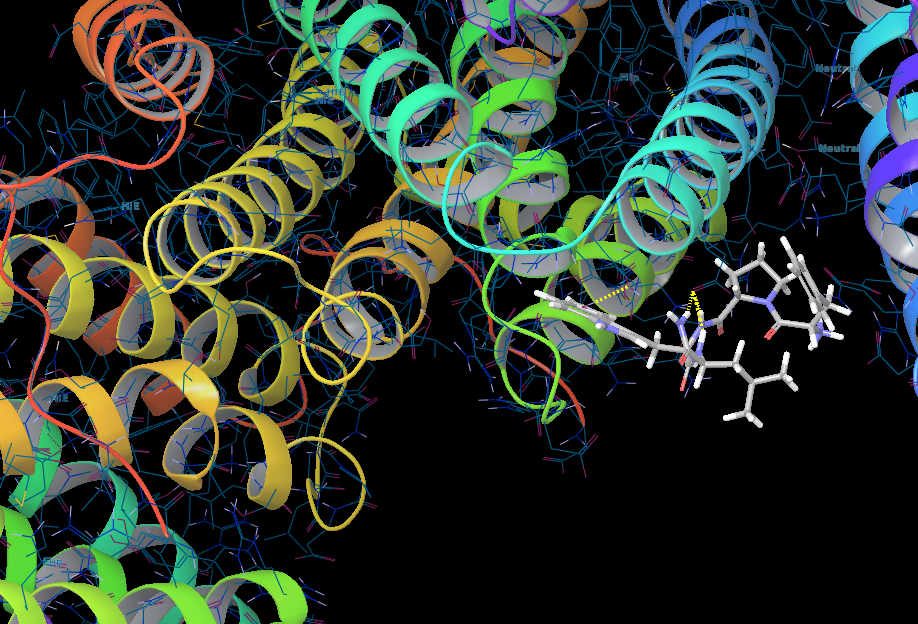

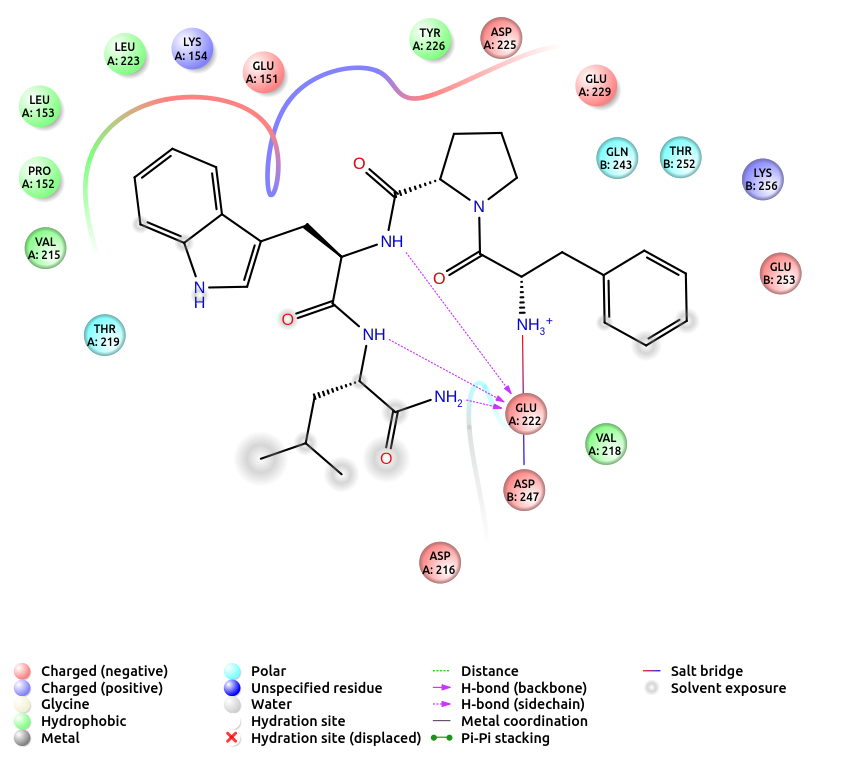


**Figure S7**: Best pose and interaction diagram for the best-docked non-GSK molecule (docking score= -7.41) for nrdF2 (Rv3048c).


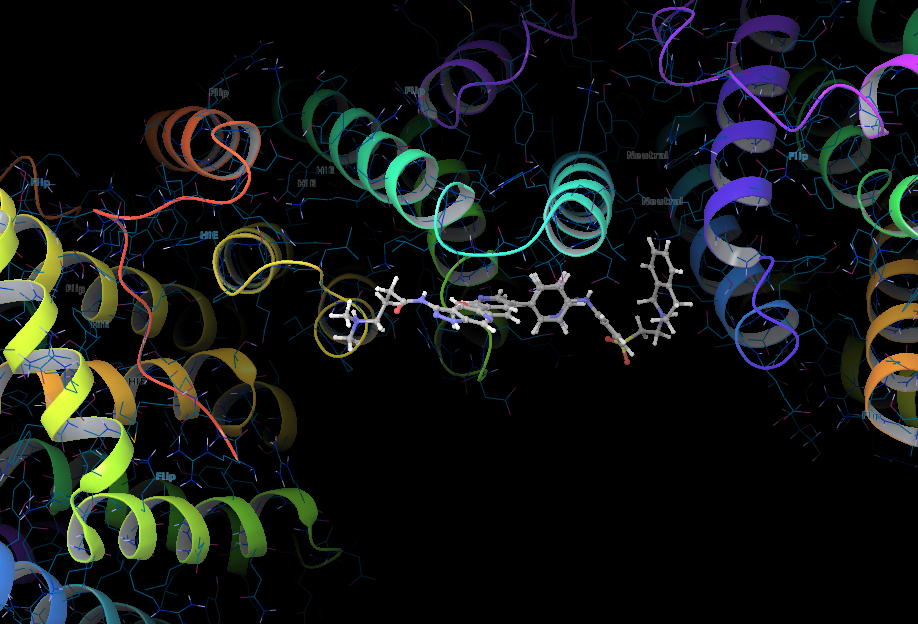

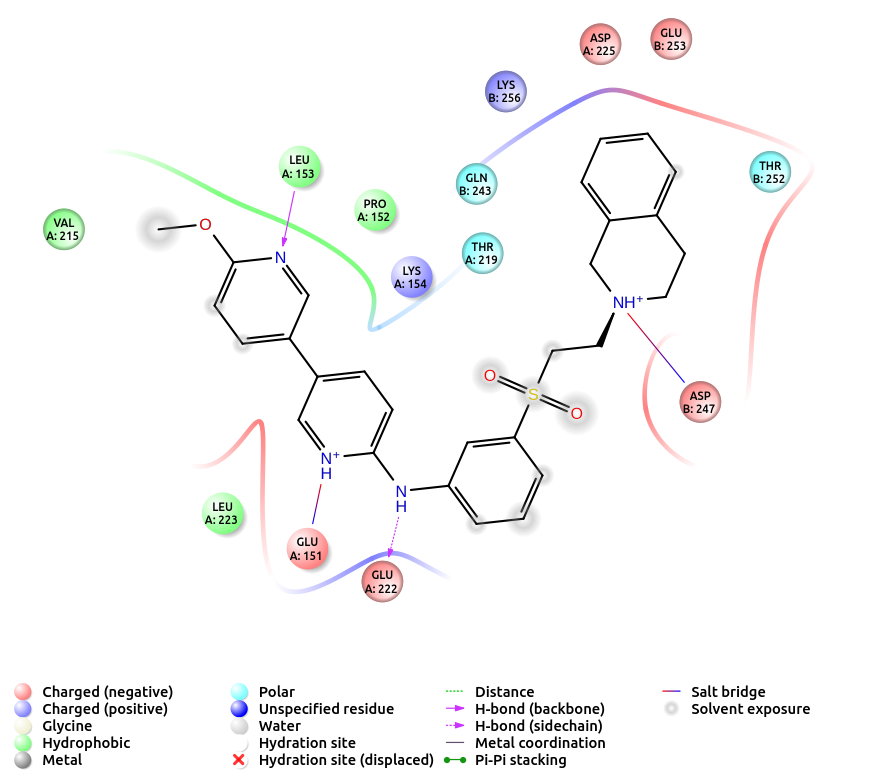


**Figure S8**: 2 Best poses with highest binding GSK compound and its interaction diagram (Docking score= -9.01) for nrdF2 (Rv3048c).

a
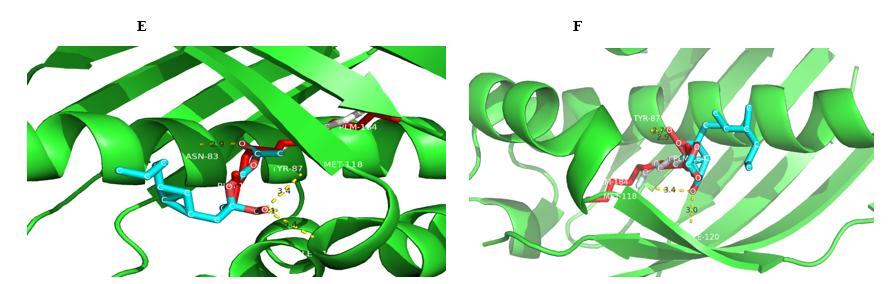
b
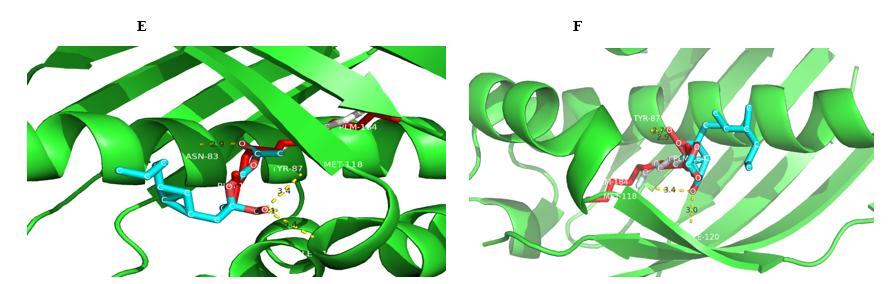


**Figure S9:** Binding pattern of two lead compounds on to the hypothetical protein (Rv0098, PDB ID 2PFC); (**a)** ChEMBL3349754, showing the hydrophobic interaction with Asn83, Met118, Lue115 and Tyr87; (**F)**ChEMBL3037996Showed hydrophobic environment around binding site residues Met 118, Ile120 and Tyr87.Red color stick showing the NS Palmitic acid –PLM and hydrogen bond interactions are represented in yellow dotted lines.


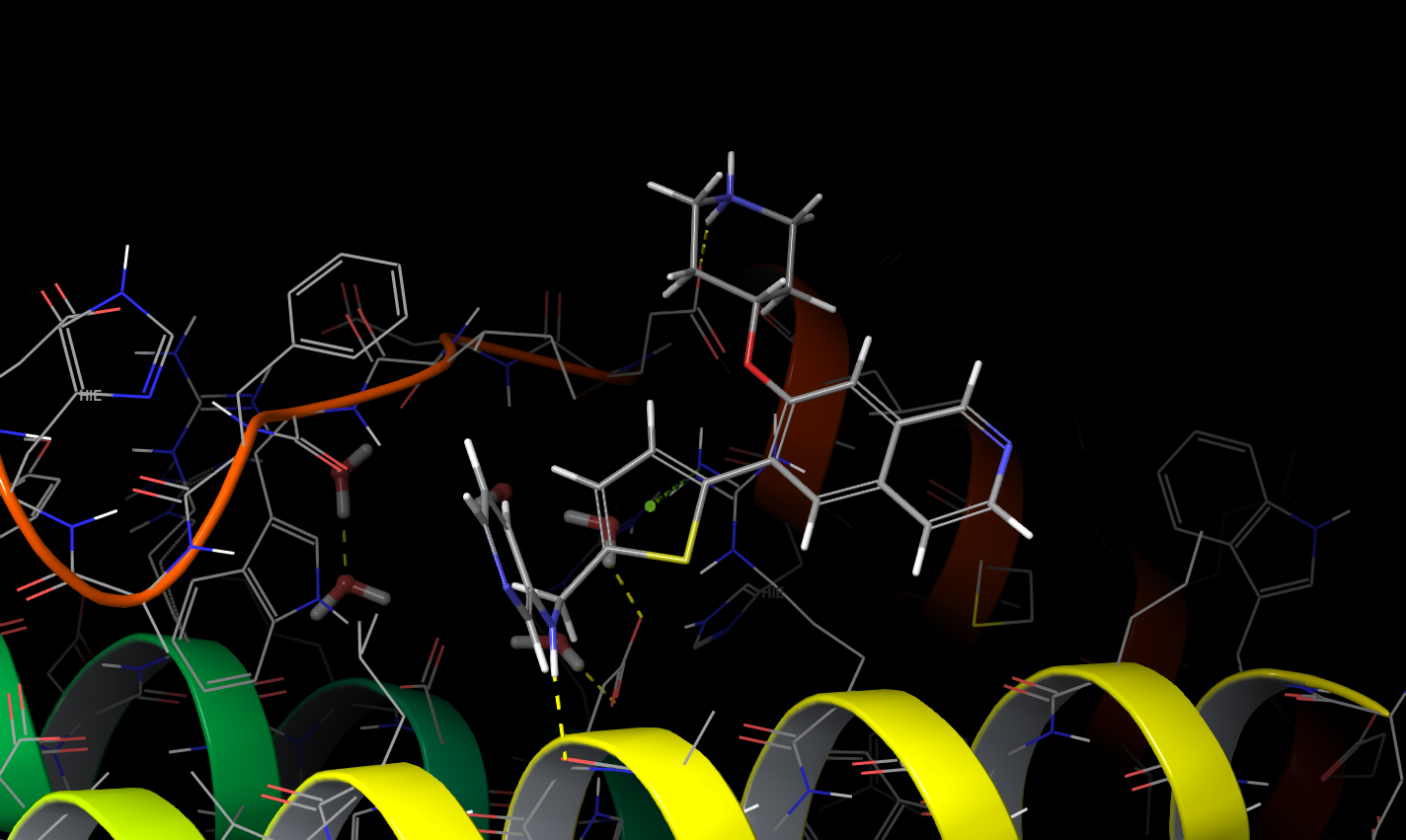

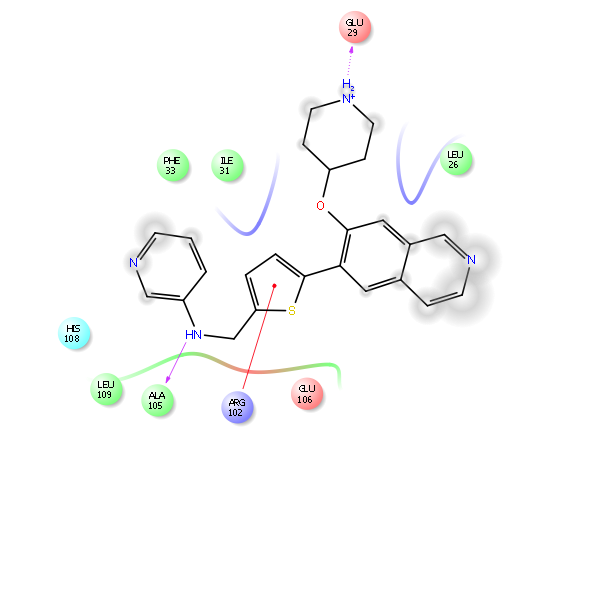


**Figure S10**: Best Docking score= -6.79 and interaction diagram obtained from the entire library of molecules for desA2 (Rv1094).


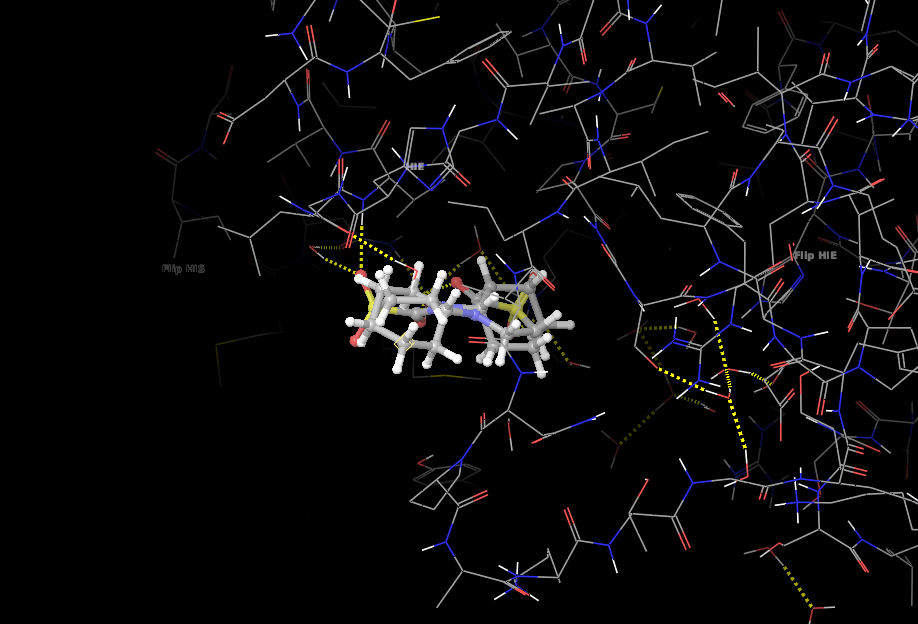


**Figure S11**:Best poses for the top ranked GSK molecule for kdtB (Rv2965c).

(a)
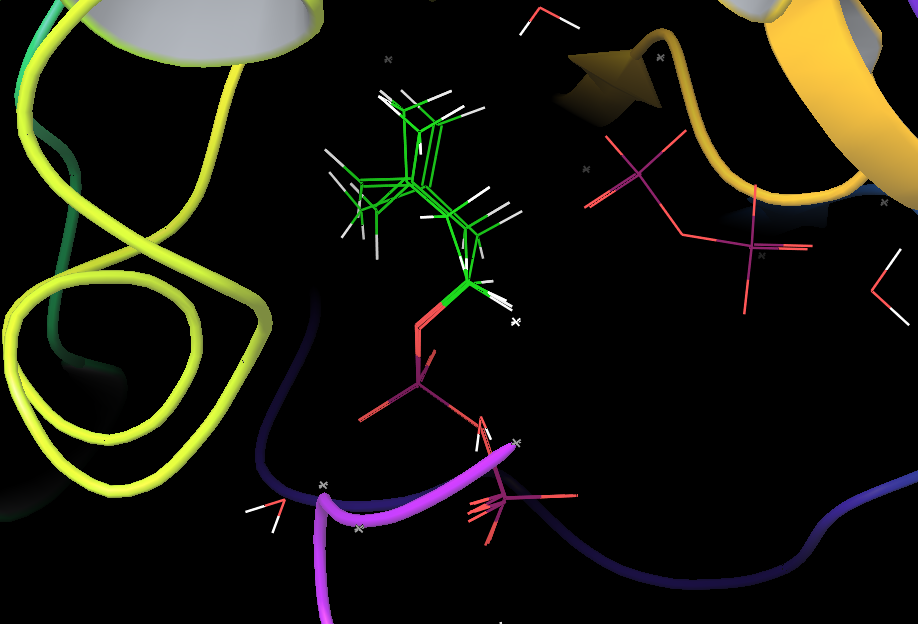
(b)
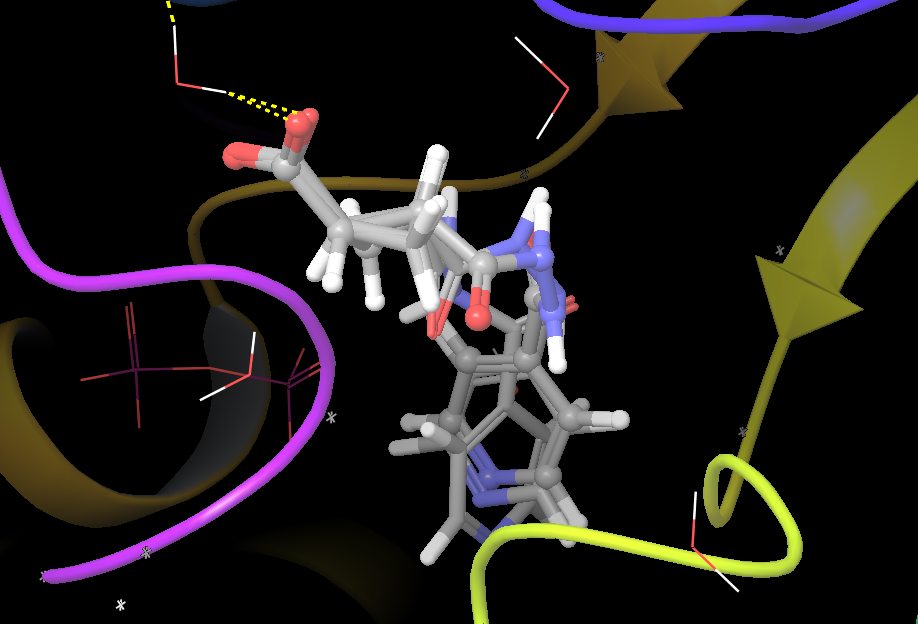


c)
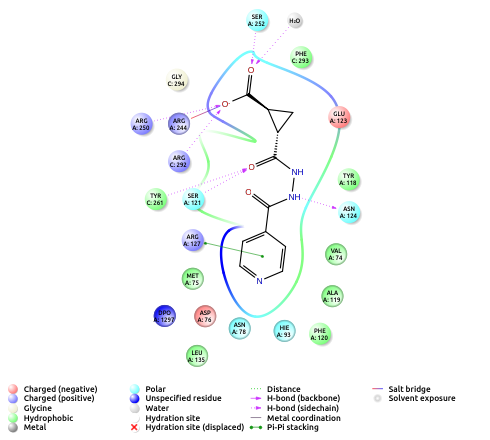


**Figure S12**: (a). Top poses of the best-docked GSK molecules, (b). Docking performed with 426 GSK molecules, (c). Interaction diagram for the best GSK molecule foruppS (Rv2361c).

a
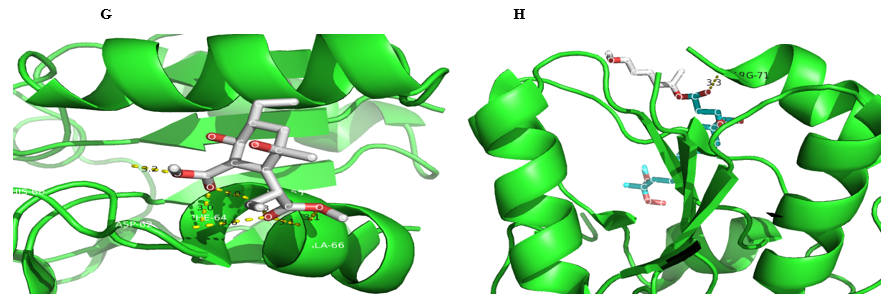
b
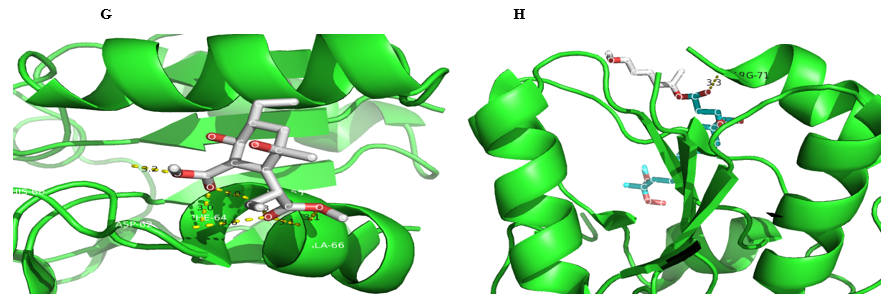


**Figure S13:** Docked complex of lead compounds into protein Rhodanese-related sulfurtransferase(Rv0390, PDB ID 2FSX);**(a)**ChEMBL217735Showed hydrophobic interaction with Phe64,His60,Ala66,Ilu65 and Asp62 represented by yellow dotted lines; (**b)** ChEMBL76817Showed hydrophobic interaction with binding site residues Arg 71are represented by yellow dotted lines


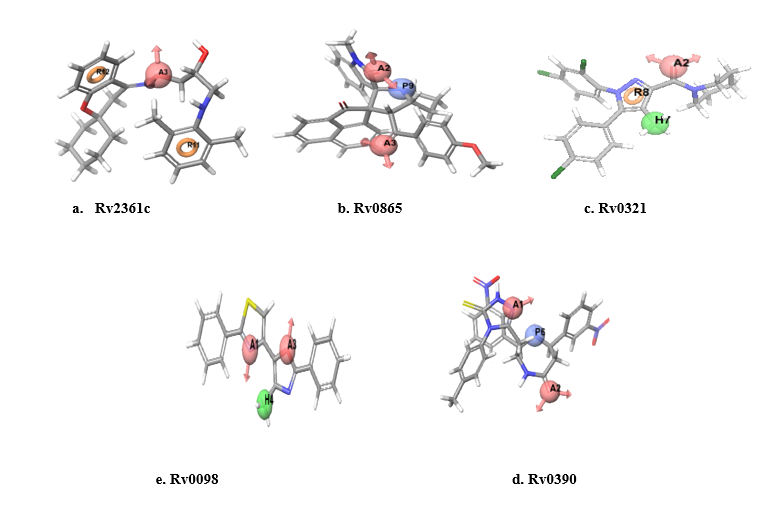


**Figure S14:**Generated Common Pharmacophore hypotheses for a set of 5 targets as: (a) ARR :( b) AAP, (c) AHR: (d) AAP: (e) AAH (green sphere/circle: hydrophobic group, orange ring: aromatic ring, Pink sphere/circle: hydrogen bond acceptor, light-blue sphere: hydrogen bond donors, blue sphere positively charged group)


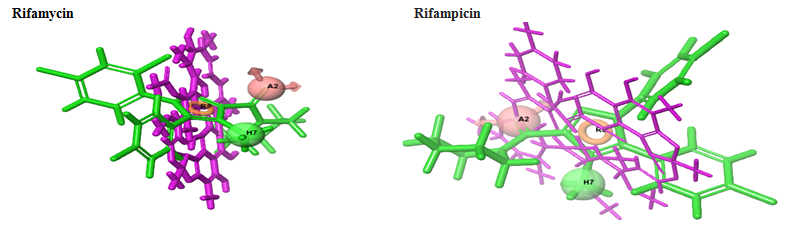


**Figure S15:**Pharmacophore feature(AHR hypothesis) mapping of external test set compounds Hydrophobic features (H7) mapped over carboxyl group, and the second hydrogen bond acceptor (A2), feature mapped over alkene group of Rifampicin with fit value of 4.74


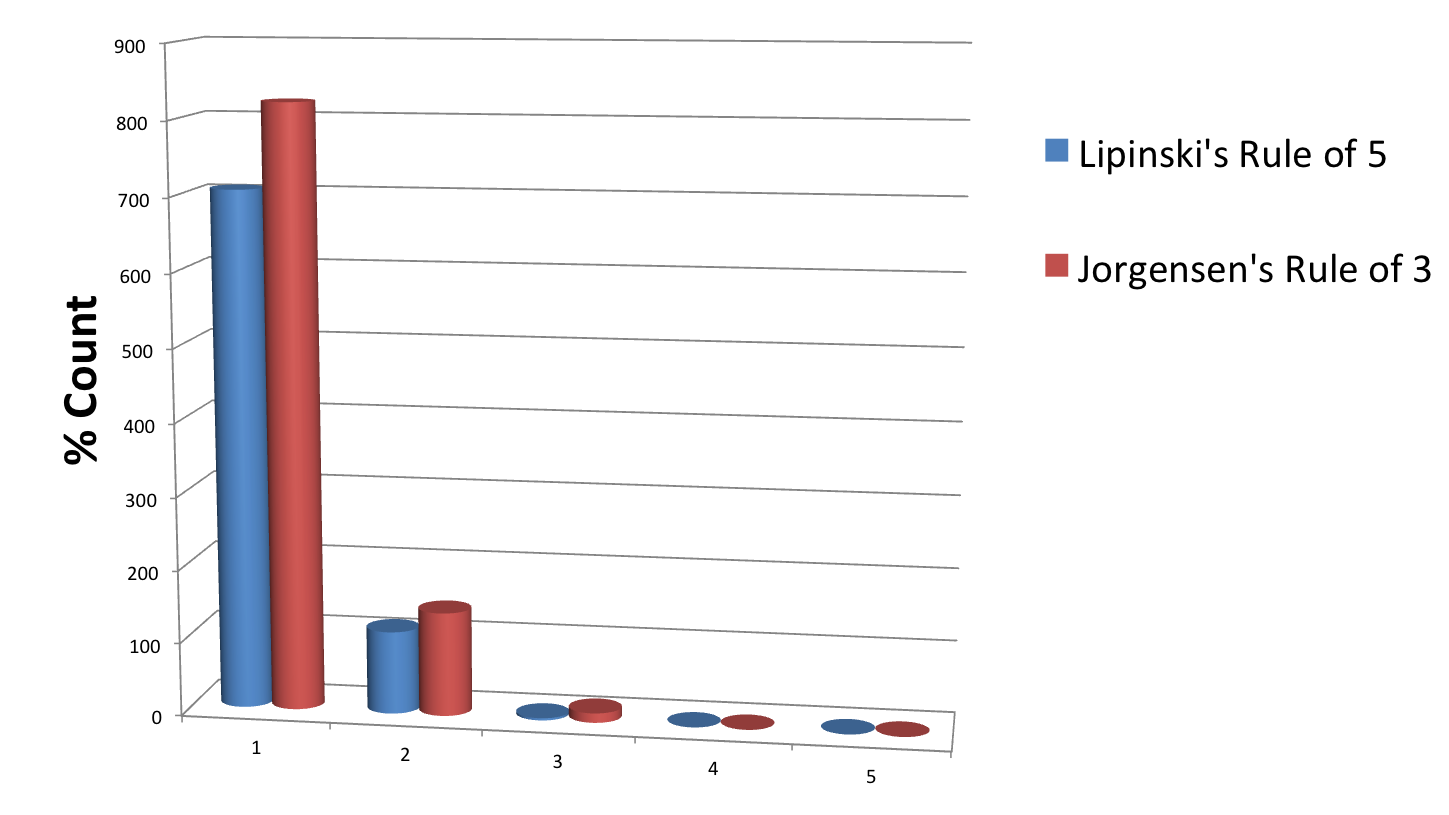


**Figure S16**: Distributions of violations of Lipinski’s ro5 and Jorgensen’s ro3 within the compound library for Rv2763c.


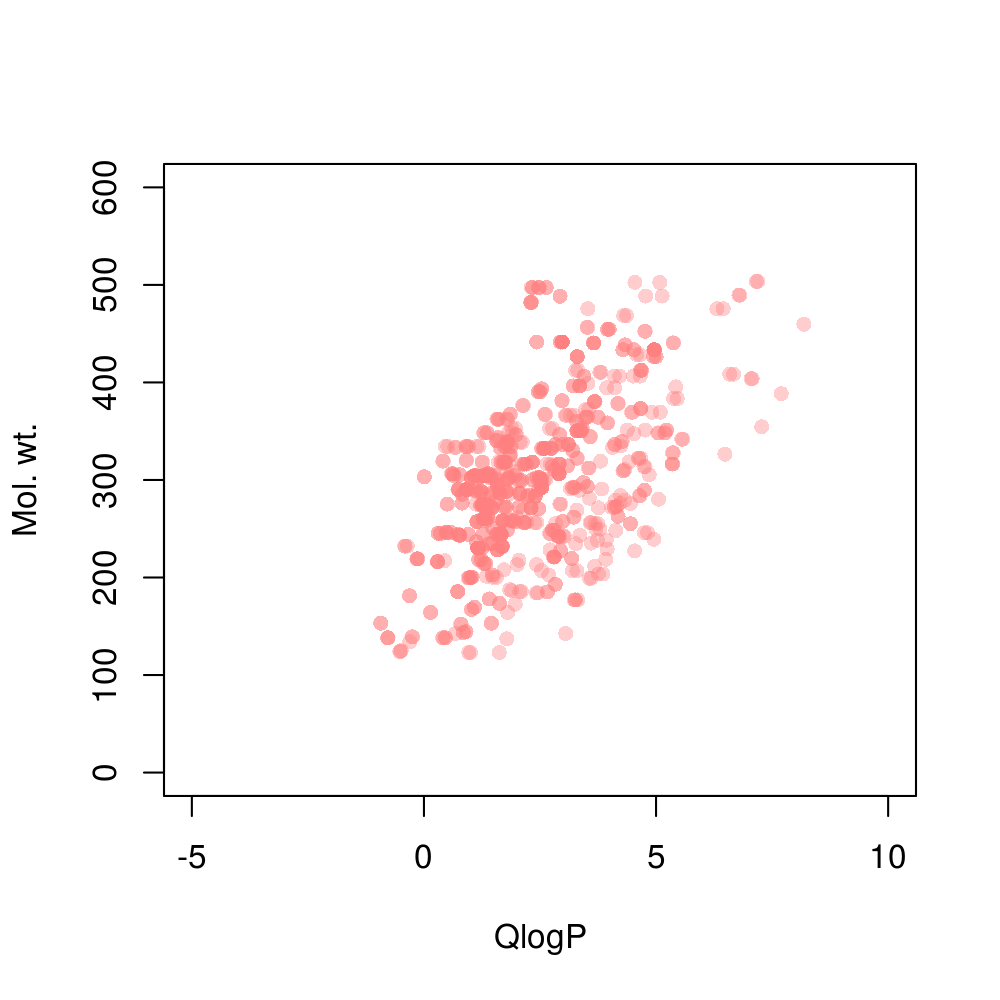


**Figure S17**: Scatter diagrams showing pair wise distribution of “drug-likeness” descriptors, MW against Predicted Octanol-Water Coefficient,QlogP for Rv2763c.


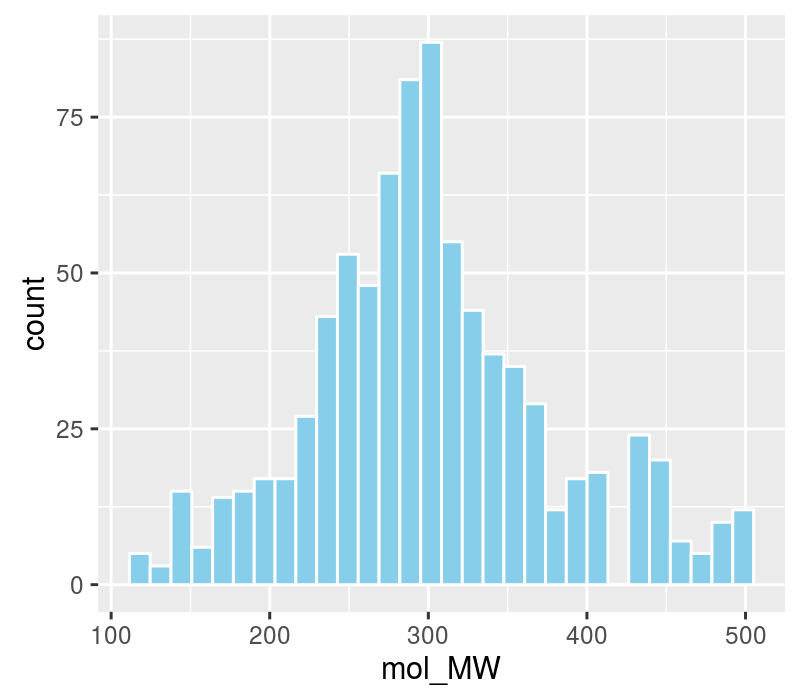

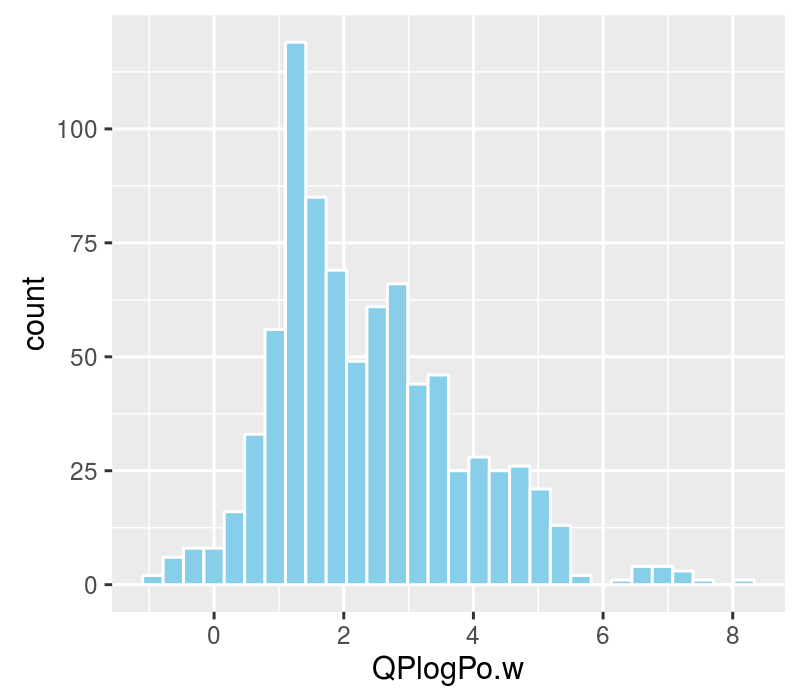


**Figure 18**: Histogram plot of the count of the compounds VsMol. Wt. and Predicted Octanol-Water Coefficient, QlogP, Drug like range: -2.0- 6.5, for Rv2763c.
